# Adaptive Hoyer-L-Moment Envelope Spectrum: A Method for Robust Demodulation of Ship-Radiated Noise in Low-SNR Environments

**DOI:** 10.3390/s25247434

**Published:** 2025-12-06

**Authors:** Ruizhe Zhang, Qingcui Wang, Shuanping Du

**Affiliations:** 1 Science and Technology on Sonar Laboratory, Hangzhou 310023, China; sklzhangrz@163.com (R.Z.); skldusp@163.com (S.D.); 2 Hangzhou Applied Acoustics Research Institute, Hangzhou 310023, China

**Keywords:** passive detection, cyclostationary analysis, spectral coherence, modulation feature extraction

## Abstract

Propeller noise is the main source of ship-radiated noise. Extracting and analyzing the modulation characteristics from the propeller noise plays a crucial role in classifying and identifying vessel targets. Existing demodulation methods such as Detection of Envelope Modulation On Noise (DEMON), narrowband demodulation, and cyclostationary analysis can be used to extract modulation features. However, capturing the modulation features on the envelope spectrum may be hard under low signal-to-noise ratio scenarios, since the envelope spectrum is contaminated by interference noise. To address this challenge, selecting an optimal frequency band rich in modulation information can significantly enhance demodulation performance. This paper proposes an Adaptive Hoyer-L-moment Envelope Spectrum (AHLES) method. The method first introduces an optimal frequency band selection method based on the golden section search strategy. A Hoyer-L-moment metric is then designed to quantify the modulation intensity within narrow frequency bands. Based on this metric, the optimal spectral coherence integration band is adaptively selected according to the signal’s inherent modulation characteristics, thereby enhancing demodulation performance. The effectiveness of the proposed method is validated through experiments on both simulated signals and merchant ship data.

## 1. Introduction

Ship-radiated noise carries abundant physical characteristics of vessels. Studying the fundamental characteristics of ship-radiated noise is essential for target identification and classification [1]. Ship-radiated noise sources can be categorized into three types: mechanical noise, propeller noise, and hydrodynamic noise. Among these, propeller noise is the most significant source of radiated noise. Cavitation noise constitutes the primary component of propeller noise, manifesting as a modulation spectrum dominated by blade rate frequency or shaft rate modulation. It contains information such as the target’s rotation speed and number of blades, serving as the main source of information for target identification. The extraction and analysis of the modulation characteristics of ship-radiated noise enable the classification and discrimination of vessel types.

**Figure 1:**
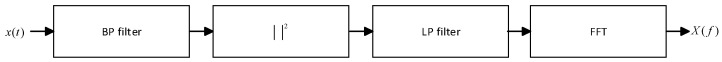
Calculation flowchart of DEMON.

Methods for extracting modulation spectrum features from ship signals can be categorized into three main types based on their principles and techniques: Detection of Envelope Modulation On Noise (DEMON) spectrum analysis, narrowband demodulation, and cyclostationary analysis. In 1988, Lourens [2] proposed DEMON analysis, a method that performs envelope demodulation on high-frequency broadband noise modulation spectra to obtain line spectral components. DEMON spectrum analysis requires several steps—bandpass filtering, detection, low-pass filtering, and spectral analysis—to derive line spectra rich in ship characteristic information. Figure 1 shows a digital DEMON analysis flow, where BP and PS stand for bandpass filtering and lowpass filtering. The selection of the demodulation bandwidth of the filter is particularly important when demodulating broadband noise. In early investigations focused on understanding the method’s performance and fundamental principles, Kudryavtsev [3] analyzed the amplitude modulation characteristics of seagoing vessels, and later Ambat [4] conducted a systematic evaluation of its performance in sonar applications. A significant advancement came with the integration of more sophisticated signal processing theories. Hanson [5] framed DEMON within the context of cyclostationarity, providing a stronger theoretical basis for passive detection. Concurrently, efforts were made to enhance the algorithm’s robustness in challenging scenarios, such as the improvements proposed by Pollara [6] to boost detection performance for small boats. In recent years, the scope of DEMON has been further extended alongside advancements in sensor technology. Stinco [7] successfully applied DEMON processing to data from an acoustic vector sensor, enabling simultaneous demodulation and direction-of-arrival estimation for multiple noise sources. This demonstrates the enduring relevance and adaptability of the DEMON technique in modern acoustic signal processing. Furthermore, some researchers [7] have defined a DEMON demodulator for Acoustic Vector Sensors (AVSs), which can acquire sound pressure and particle velocity to extract multiple modulation signals and measure their directions of arrival. These studies have significantly enhanced the performance of DEMON analysis, establishing it as one of the mainstream demodulation methods.

Since acquired modulation signals are often contaminated by significant background noise and interference, a filtering operation is typically required prior to envelope demodulation to improve the Signal-to-Noise Ratio (SNR) of the processed signal. The technique corresponding to the above procedure is referred to as narrowband envelope demodulation. More specifically, the implementation of narrowband envelope demodulation involves three steps: frequency band decomposition, demodulation band selection, and envelope demodulation analysis. While the implementation of envelope demodulation analysis is relatively standardized, the processes of frequency band decomposition and demodulation band selection are critical to the performance of narrowband envelope demodulation. Therefore, narrowband envelope demodulation can be conceptually regarded as a focused investigation into the critical BP filter selection step within the classical DEMON framework. As a result, researchers have focused on improving these two aspects. Antoni proposed a fast computation method for the Kurtogram based on a 1/3-binary tree structured filter bank [8], which significantly enhanced the efficiency of optimal demodulation band selection and was validated using several gear and bearing fault cases. Since then, the fast Kurtogram algorithm has gradually become a mainstream method for narrowband envelope demodulation. In subsequent studies, scholars introduced several improved metrics and narrowband demodulation methods based on the spectral kurtosis index. Considering the impulsivity and cyclostationarity of modulation signals from mechanical faults in moving parts, Antoni [9] proposed Infogram, a demodulation band selection method based on an information entropy metric. To overcome the limitations of spectral kurtosis under low-SNR conditions and non-Gaussian noise interference, Moshrefzadeh [10] introduced the Autogram, a demodulation band selection method based on the kurtosis value of the autocorrelation of the envelope signal.

As an emerging technology in the field of information processing, cyclostationarity theory and methods began to be applied around the 1990s. Initially proposed and studied in communications to meet the needs of sonar, radar, and remote sensing [11,12], it was introduced into mechanical condition monitoring around the year 2000. Over the past two decades, cyclostationarity theory has been thoroughly and deeply investigated [13,14,15,16] and widely applied in fault diagnosis of various machinery types [17,18]. Essentially, the periodic operation of rotating machinery implies that any such machine can be modeled using cyclostationary frameworks and analyzed with corresponding cyclostationary tools based on their cyclic characteristics.

The Vibrations and Acoustics Laboratory at the University of Lyon has conducted extensive work on modulation feature extraction based on cyclostationarity. For example, in 2000, their research revealed that rolling bearing fault signals exhibit second-order cyclostationarity and clarified the mathematical relationship between envelope demodulation and cyclostationary analysis, demonstrating that the latter offers higher demodulation accuracy [19]. In 2004, they investigated the relationship between angle-time cyclostationarity and established a cyclostationary model for rotating machinery signals [20]. In 2005, they proposed a blind source extraction method via cyclic statistics and subspace decomposition [13] and introduced an averaged cyclic periodogram estimator to address spectral leakage in smoothed cyclic periodograms [21]. In 2007, they proved the relationship between the cyclic spectral correlation/coherence and the squared envelope spectrum, and further studied estimation issues in cyclic spectral coherence analysis [15,22].

Although spectral correlation offers excellent demodulation performance, classical unbiased estimators such as the Averaged Cyclic Periodogram (ACP) suffer from high computational complexity, making them impractical for real-time industrial monitoring. Meanwhile, other rapid estimators like the Cyclic Modulation Spectrum (CMS) and the FFT Accumulation Method (FAM) are biased [23]. To overcome these limitations, Antoni proposed two fast cyclostationary algorithms in 2017 and 2018, which were validated using publicly available bearing data from Case Western Reserve University, demonstrating both effectiveness and efficiency [23,24]. These fast spectral correlation algorithms based on short-time Fourier transform have essentially removed the major obstacles for engineering applications of cyclostationarity.

Recently, how to choose the optimal integration frequency band for spectral correlation or coherence has become an emerging research topic. Wang [25] proposed using the ratio of the 2-norm to the 1-norm to select the carrier frequency band richest in information, followed by integrating the spectral coherence within the chosen band. Mauricio [26,27] utilized cyclostationarity to measure the amount of modulation information within carrier frequency bands and introduced the IESFOgram for selecting the optimal integration band. Both methods, however, require prior knowledge of the target characteristic frequency. To address this, Lee [28] computed the frequency-domain autocorrelation function at specific cyclic frequencies and applied weighting to the spectral coherence, proposing the Weighted Enhanced Envelope Spectrum (WEES). Building on this, Tong [29] adaptively assigned weighting coefficients to the spectral coherence based on the modulation intensity of each spectral component, leading to the Adaptive Weighted Envelope Spectrum (AWES). Although such weighted methods eliminate the need for prior knowledge, they assume that the cyclostationary signal of interest is dominant. When cyclostationary noise—such as electromagnetic interference or signals from other mechanical components—dominates, these methods may fail to extract the target frequency.

In this paper, an Adaptive Hoyer-L-moment Envelope Spectrum (AHLES) is pro-posed for detecting ship target modulation signals. The primary contributions of this work are summarized in two aspects:

(1) A Hoyer-L-moment (HL) metric is proposed to evaluate the modulation intensity of individual spectral component from both sparsity and periodicity, without requiring prior knowledge.
(2) A Golden Ratio Band Division (GRBD) method is proposed to adaptively divide the frequency spectrum and select the optimal integration bands. Based on the golden section search principle, GRBD efficiently partitions the frequency band and identifies the integration bands with the most intense modulation characteristics.

The structure of this paper is organized as follows: Section 2 discusses the principles of cyclostationary analysis. Section 3 details the AHLES methodology, including the GRBD method and the design of the HL metric. Section 4 validates the proposed method using simulated signals and merchant ship data, providing comparative experimental results. Section 5 concludes the paper.

## 2. Cyclostationary Analysis

The spectral correlation (SC) and spectral coherence (SCoh) of cyclostationary signals are essential tools for analyzing propeller modulation signals.

For a non-stationary signal x(t), if its time-varying autocorrelation function $R_{x}\left(t,\tau \right)$ is periodic in t with period T, then x(t) is second-order cyclostationary [22]. The $R_{x}\left(t,\tau \right)$ can be expressed as

(1) $$R_{x}\left(t,\tau \right)=E\left\{x^{*}\left(t-\frac{\tau }{2}\right)x\left(t+\frac{\tau }{2}\right)\right\}=R_{x}\left(t+T,\tau \right)$$

where $x^{*}\left(\cdot \right)$ represents the complex conjugate of $x\left(\cdot \right)$, τ is the time delay, and $E\left\{\cdot \right\}$ indicates the statistical averaging.

The $S_{x}(\alpha ,f)$, standing for SC defined as the Fourier transform of the instantaneous autocorrelation function, can be derived as

(2) $$S_{x}\left(\alpha ,f\right)=\frac{1}{T}\sum_{-T/2}^{T/2}\sum_{-\infty }^{\infty }R_{x}\left(t,\tau \right)e^{-j2\pi \alpha t}e^{-j2\pi f\tau }$$

where f denotes the spectral frequency and α represents the cyclic frequency. For second-order cyclostationary signals, the $S_{x}(\alpha ,f)$ is a bivariate spectrum which exhibits continuity in spectral frequency f and discreteness in cyclic frequency α and it can be reformulated as

(3) $$S_{x}\left(\alpha ,f\right)=\left\{\begin{array}{c} S_{x}^{k}\left(f\right),\alpha =k/T\\ 0,\;\mathrm{else} \end{array}\right.$$

where $S_{x}^{k}(f)$, k=0,±1,±2,… represents the cyclic spectra [16].

Furthermore, to mitigate the effects of non-uniform noise distribution, the SCoh is defined as follows [16]:

(4) $$\gamma_{x}\left(\alpha ,f\right)=\frac{S_{x}\left(\alpha ,f\right)}{\sqrt{S_{x}\left(0,f\right)\cdot S_{x}\left(0,\alpha +f\right)}}$$

Equation (4) serves as a statistical measure of the strength of cyclostationarity at a specific frequency pair. When $\left|\gamma_{x}\left(\alpha ,f\right)\right|=1$, the signal x(t) is considered fully coherent at spectral frequency f and cyclic frequency α; when $\left|\gamma_{x}\left(\alpha ,f\right)\right|=0$, it indicates that x(t) is completely incoherent at spectral frequency f and cyclic frequency α.

Based on Equation (4), the Enhanced Envelope Spectrum (EES) can be derived as follows [23]:

(5) $$S_{x}^{EES}\left(\alpha \right)=\int_{f_{1}}^{f_{2}}\left|\gamma_{x}\left(\alpha ,f\right)\right|df$$

where $f_{2}-f_{1}$ denotes the selected integration bandwidth.

## 3. The Methodology of Adaptive Hoyer-L-Moment Envelope Spectrum

The bi-spectrum of Scoh contains information on both the carrier and modulation frequencies. However, its two-dimensional nature renders it impractical for most engineering applications, where one-dimensional spectral analysis is preferred due to simplicity; hence, the EES has been widely adopted in both academic and industrial practice. Nevertheless, under low-SNR conditions, modulation components are often obscured by noise, and integrating over the entire Nyquist band may fail to capture weak modulation. To address this limitation, integrating over specific frequency bands rich in modulation energy can effectively enhance the envelope spectrum and improve the detection performance of modulation frequencies.

### 3.1. Golden Ratio Band Division

**Figure 2:**
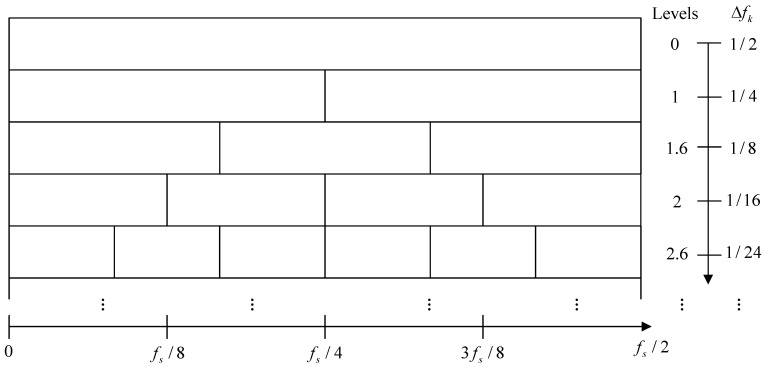
1/3-binary tree structure.

Demodulation frequency band division is the first step in identifying the optimal integration frequency band, and a reasonable band division structure is crucial to this process. To accommodate various operating conditions—such as different SNRs and varying degrees of harmonic interference—the band division structure should possess the ability to adaptively adjust the bandwidth of sub-bands, thereby obtaining a set of frequency bands with minimal interference and enhancing the effectiveness and robustness of demodulation analysis techniques.The most commonly used band division structure is the 1/3-binary tree, as illustrated in Figure 2. However, the 1/3-binary tree structure has limitations, such as the inability to access certain frequency segments. For example, when the center frequency of the demodulation band is $f_{c}=f_{s}/4,\;f_{s}/8,\;f_{s}/16,\;\ldots ,\;3f_{s}/8$, the 1/3-binary tree structure may fail to simultaneously capture the adjacent frequency band groups on both sides of the center frequency.

Furthermore, the determination of the maximum decomposition level *k* generally relies on empirical experience, meaning that the 1/3-binary tree structure cannot adaptively balance computational cost and frequency band division accuracy.

In addition, the spectral frequency resolution Δf of the Fast-SC algorithm is determined by the sampling frequency $f_{s}$ and the short-time Fourier transform window length $N_{w}$ as

(6) $$\Delta f=\frac{f_{s}}{N_{w}}$$

As can be seen from Equation (6), due to the typically high sampling rate $f_{s}$ of propeller cavitation signals and the small value of the window width $N_{w}$, the spectral frequency resolution Δf is generally poor.

Furthermore, the number of discrete frequency points $N_{n}$ can be expressed as

(7) $$N_{n}=N_{w}/2$$

In the Fast-SC algorithm, $N_{w}$ is typically set to a power of two to facilitate efficient computation. Therefore, within the 1/3-binary tree structure, the number of discrete spectral frequencies $\rho_{k}$ at each decomposition level *k* can be defined as

(8) $$\rho_{k}=\frac{N_{n}}{2^{k}\Delta f}$$

where $\rho_{k}$ denotes the number of discrete spectral frequencies at decomposition level *k*. Since $N_{n}$ may not be divisible at fractional decomposition levels such as k=0.6,1.6,2.6,…, $\rho_{k}$ may not be an integer.

**Figure 3:**
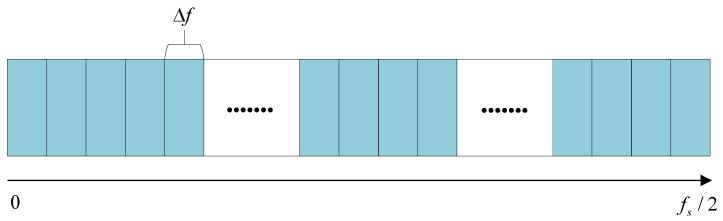
Divided structure.

Moreover, empirical observations indicate that the 1/3-binary tree structure tends to select frequency bands at the maximum decomposition level. As a result, computations at lower decomposition levels are redundant. Additionally, given the generally poor spectral frequency resolution Δf in the Fast-SC algorithm, it is feasible to perform frequency band division directly based on Δf, as illustrated in Figure 3.

Conventional frequency band division methods typically select the band with the strongest modulation intensity for SCoh integration. However, in practice, the spectral structure is complex and can be composed of multiple carrier bands with variable bandwidths and non-uniform distributions. Using a single fixed band for SCoh integration often fails to achieve optimal demodulation performance.

To address this limitation, this paper proposes a frequency band division method based on the golden section strategy, referred to as the Golden Ratio Band Division (GRBD). This method adaptively selects bands with high modulation intensity according to the signal’s inherent modulation characteristics, thereby identifying the optimal demodulation frequency band. The specific procedure is as follows:

Step 1: Divide the Nyquist frequency band along the frequency axis f into sub-bands with the width Δf, where sub-bands is [iΔf,(i+1)Δf], $i=0,1,2,\ldots ,N_{n}-1$.

Step 2: Based on the lower and upper cutoff frequencies iΔf and (i+1)Δf of the divided sub-bands, perform integration along the spectral frequency f to obtain the EES for each sub-band:

(9) $$S_{x}^{EES_{i}}\left(\alpha \right)=\int_{i\Delta f}^{(i+1)\Delta f}\left|\gamma_{x}\left(\alpha ,f\right)\right|df$$

Step 3: Define HL as an indicator value representing the modulation intensity of each sub-band. A higher HL value indicates richer modulation information. Compute the HL indicator value for the EES of each sub-band.

Step 4: Set the initial interval for optimizing the scale factor σ as [a,b]=[0,1]. Calculate the initial scaling factors $\sigma_{1}^{0}=a+(1-\rho )(b-a)$ and $\sigma_{2}^{0}=a+\rho (b-a)$, where $\rho =(\sqrt{5}-1)/2$. Then, rank all sub-bands in descending order based on their HL values. From this sorted sequence, select the top $M_{\left(k,i\right)}$ sub-bands $B_{j}^{\left(k,i\right)}$ where $M_{\left(k,i\right)}=floor(\sigma_{i}^{k}N_{n})$, $j=1,2,\ldots ,M_{\left(k,i\right)}$, floor(⋅) denotes the rounding-down operation. And $\sigma_{i}^{k}$ represents the *i*th scaling factor in the *k*th iteration, where i=1,2, k=0,1,2,3,….

Step 5: Calculate the EES corresponding to $\sigma_{i}^{k}$: $S_{x}^{\mathrm{EES}}(\alpha ;\sigma_{i}^{k})=\sum_{j=1}^{M}\int_{f(j)}^{f(j)+\Delta f}\left|\gamma_{x}\left(\alpha ,f\right)\right|df$, where f(j) is the starting frequency of sub-band $B_{j}^{\left(k,i\right)}$.

Step 6: Compute the value $X_{i}^{k}=HL(\sigma_{i}^{k})$ for $\sigma_{i}^{k}$.

Step 7: Optimize $\sigma_{i}^{k}$ using the golden section method. Update the interval $\left[a_{k},b_{k}\right]$ and scaling factors $\sigma_{1}^{k}$, $\sigma_{2}^{k}$ iteratively as follows:

(10) $$\mathrm{While}\left|{X_{best}}^{k}-{X_{best}}^{k-1}\right|>\epsilon :\;\left\{\begin{array}{l} \mathrm{If}\;{X_{1}}^{k}<{X_{2}}^{k}:\\ \;a_{k}={\sigma_{1}}^{k},{\sigma_{1}}^{k}={\sigma_{2}}^{k},{X_{1}}^{k}={X_{2}}^{k}\\ \;{\sigma_{2}}^{k+1}=a_{k}+\rho (b_{k}-a_{k}),{X_{2}}^{k+1}=HL({\sigma_{2}}^{k+1})\\ \mathrm{Else}:\\ \;b_{k}={\sigma_{2}}^{k},{\sigma_{2}}^{k}={\sigma_{1}}^{k},{X_{2}}^{k}={X_{1}}^{k}\\ \;{\sigma_{1}}^{k+1}=a_{k}+(1-\rho )(b_{k}-a_{k}),{X_{1}}^{k+1}=HL({\sigma_{1}}^{k+1}) \end{array}\right.$$

where ϵ denotes the interval tolerance and ${X_{best}}^{k}=\max\{{X_{1}}^{k},{X_{2}}^{k}\}$. The iteration stops when $\left|{X_{best}}^{k}-{X_{best}}^{k-1}\right|\leq \epsilon$. In this study, to balance computational efficiency and accuracy, the interval tolerance is set to $\epsilon =10^{-4}$. The optimal $\sigma_{best}$ corresponds to $X_{best}^{k}$, and the optimal demodulation frequency band $B_{j}(\sigma_{best})$ is output.

**Figure 4:**
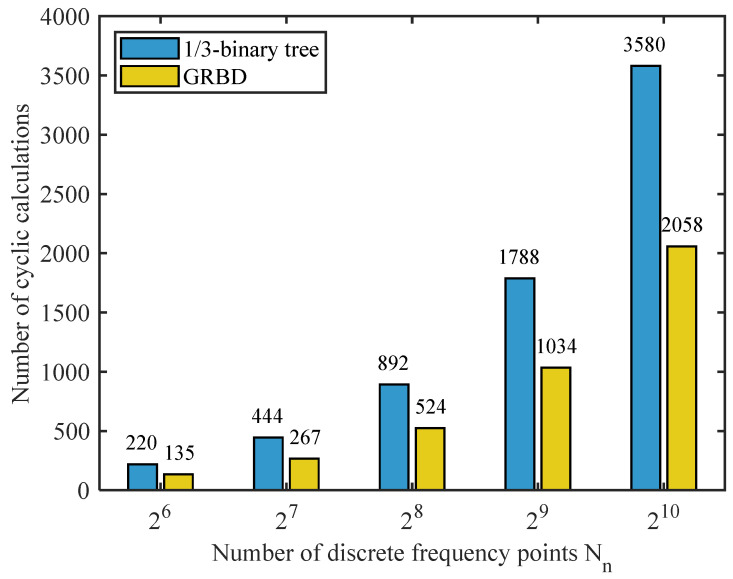
Computational cost of two frequency band division structures.

To compare the computational cost of the GRBD method and the 1/3-binary tree structure, the following modulated signal model is used for simulation analysis:

(11) $$x(t)=[1+\sum_{i=1}^{p}A_{i}\cos(2\pi k\alpha t)]\cdot c(t)+n_{s}(t)$$

where the modulation frequency is set to *α* = 5 Hz, the number of harmonics is *p* = 4, *A_i_* = 1, the broadband carrier c(t) is located in the frequency ranges of 2–3 kHz and 5–6 kHz, the data length is 10 s, the sampling rate is 20 kHz, and the SNR is set to −10 dB. To effectively compare the computational costs of the two frequency band division structures, the minimum bandwidth of the 1/3-binary tree structure is set to Δf. Figure 4 shows the computational cost of the two methods, measured by the number of cyclic calculations. The GRBD method not only exhibits a lower absolute computational cost across all $N_{n}$, but also a slower growth rate of computational complexity as $N_{n}$ increases. This demonstrates the superior computational efficiency of the GRBD method.

### 3.2. Hoyer-L-Moment Metric

Selecting an appropriate metric to evaluate the richness of modulation information contained within each sub-band is also a key factor in choosing the integration frequency band. A commonly used evaluation metric is the kurtosis of the envelope spectrum, defined as follows:

(12) $$K_{e}=\frac{E\left({\left|H_{e}\left(f\right)\right|}^{4}\right)}{{\left(E\left({\left|H_{e}\left(f\right)\right|}^{2}\right)\right)}^{2}}-2$$

where $K_{e}$ denotes the spectral kurtosis and $H_{e}$ represents the envelope spectrum.

However, kurtosis only quantifies sparsity and cannot capture periodicity. Signals with multiple harmonics may exhibit low kurtosis values, which contradicts expectations. Therefore, an effective evaluation metric must account for both the periodicity and the sparsity of the envelope spectrum. In this study, we propose a new metric, Hoyer-L-moment (HL), which combines the advantages of Hoyer index and L-moments to evaluate the sparsity and periodicity of the spectrum, thereby assessing the modulation components in the frequency band decomposition results.

L-moments, defined as a series of linear functions of the expectations of order statistics, provide an effective tool for characterizing both the periodicity and sparsity of line spectra [30]. Assume that $X=[X_{1},X_{2}\ldots ,X_{n}]$ is a set of continuous independent samples of size *n*, drawn from a cumulative distribution F(x), and let $X_{1:n}\leq X_{2:n}\leq \cdot \cdot \cdot \leq X_{n:n}$ denote the order statistics of the random variables drawn from *X*. The *r*th L-moment $\lambda_{r}$ of the independent sample *X* is calculated as follows:

(13) $$\lambda_{r}=\frac{1}{r}\sum_{k=0}^{r-1}{\left(-1\right)}^{k}\left(\begin{array}{c} r-1\\ k \end{array}\right)E(X_{r-k:r}),\;r=1,2,\ldots$$

where $E(X_{r-k:r})$ denotes the expectation of the order statistic $X_{r-k:r}$, defined as

(14) $$E(X_{j:r})=\frac{r!}{(j-1)!(r-j)!}\int_{0}^{1}x{\left[F(x)\right]}^{j-1}{\left[1-F(x)\right]}^{r-j}dF(x)$$

Based on this concept, the L-moment ratios (*L*-skewness and *L*-kurtosis) are expressed as

(15) $$L-\mathrm{skewness}=\frac{\lambda_{3}}{\lambda_{2}}=\frac{2E(X_{3:3}-2X_{2:3}+X_{1:3})}{3E(X_{2:2}-X_{1:2})}$$

(16) $$L-\mathrm{kurtosis}=\frac{\lambda_{4}}{\lambda_{2}}=\frac{E(X_{4:4}-3X_{3:4}+3X_{2:4}-X_{1:4})}{2E(X_{2:2}-X_{1:2})}$$

Compared to traditional skewness and kurtosis, *L*-skewness and *L*-kurtosis exhibit less bias toward outliers and are more robust, especially under data-scarce conditions. This is because L-moment ratios are linear functions of order statistics, which significantly mitigate the influence of outliers. In contrast, traditional skewness and kurtosis rely on the third and fourth moments of samples, respectively, which inevitably amplify the weight of outliers, leading to substantial bias and variance.

The Hoyer index is a sparsity metric used to quantify the concentration of energy distribution in a signal. It is defined as:

(17) $$H=\frac{\sqrt{n}-\|x{\|}_{1}/\|x{\|}_{2}}{\sqrt{n}-1}\;$$

where $\left\|x{\|}_{1}=\sum_{i=1}^{n}|x_{i}\right|$ is the L1 norm reflecting the degree of energy dispersion in the signal, and $\|x{\|}_{2}=\sqrt{\sum_{i=1}^{n}x_{i}^{2}}$ is the L2 norm representing the uniformity of amplitude distribution. When the signal has only one non-zero element, the Hoyer index equals 1 (fully sparse). When the signal elements are uniformly distributed, the Hoyer index equals 0 (non-sparse).

Studies have shown that the Hoyer index remains effective in measuring high sparsity even under multi-harmonic and low-SNR conditions [31]. L-moments are metrics that quantify the statistical characteristics of periodic pulses. Since the Hoyer index measures the concentration of signal energy, combining these two indicators provides an effective measure of periodic sparse pulses. This composite metric is defined as

(18) $$HL=\frac{\sqrt{n}-\|x{\|}_{1}/\|x{\|}_{2}}{\sqrt{n}-1}\times \left|\frac{\lambda_{3}}{\lambda_{2}}\right|\times \frac{\lambda_{4}}{\lambda_{2}}$$

**Figure 5:**
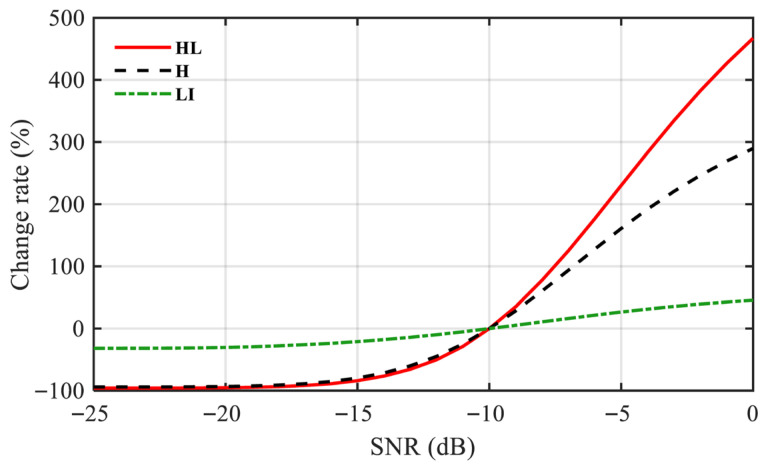
The relative change rates of three indicators with different SNR.

The *HL* metric inherits the advantageous properties of both the L-moments and the Hoyer index, exhibiting high sensitivity to periodic signals and strong robustness against noise interference. To evaluate the performance of these three metrics under different SNR conditions, a Monte Carlo experiment was conducted. The simulation signal parameters are consistent with those in Section 3.1, and the experiment was repeated 1000 times. The average rate of change in the metrics of the EES was compared, using the values at −10 dB as the baseline. As shown in Figure 5, compared to using either metric alone, the combined indicator HL demonstrates superior sensitivity to modulation information in low-SNR scenarios.

**Figure 6:**
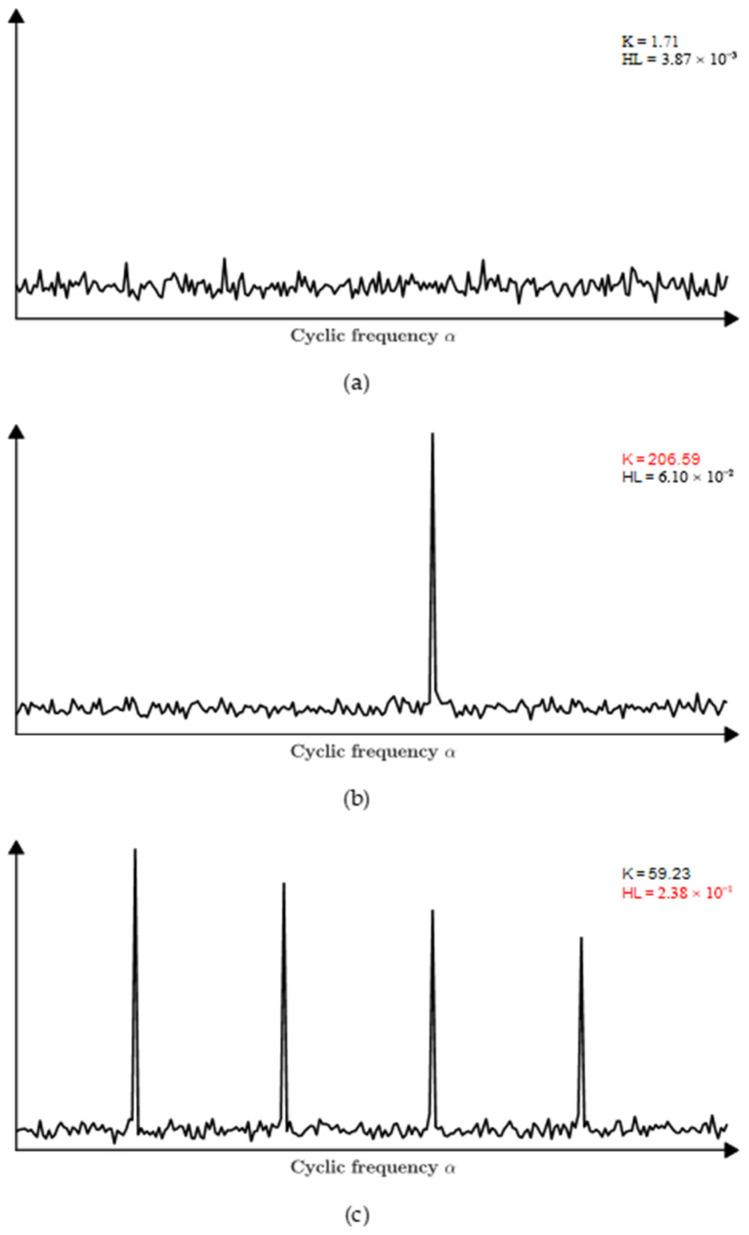
Kurtosis and HL values for several spectrum slices: (**a**) spectrum slice outside the carrier frequency; (**b**) spectrum slice with a peak of interest; (**c**) spectrum slice with four peaks of interest.

Moreover, HL is compared with kurtosis in terms of its capability to measure periodicity. As shown in Figure 6, unlike kurtosis—which tends to peak under the influence of a single impulse—the HL approaches its maximum value when periodic impulses occur consistently around specific cyclic frequencies. This indicates that the HL is more suitable for evaluating the demodulation strength of signals with periodic modulation patterns.

In summary, the HL demonstrates superior performance over traditional kurtosis, as well as individual L-moments or the Hoyer index alone, in capturing periodic sparsity, which is more suitable for identifying and quantifying modulation intensity in frequency bands.

### 3.3. Modulation Feature-Extraction-Based AHLES

**Figure 7:**
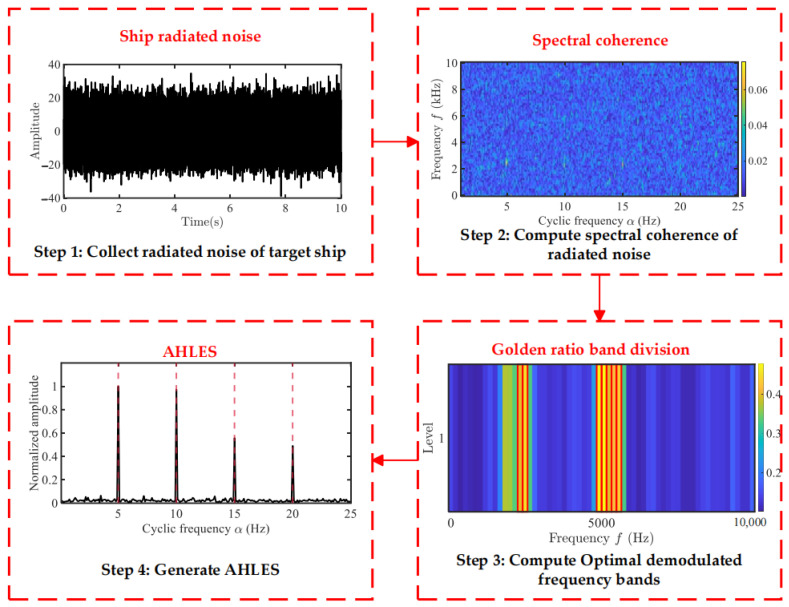
Flowchart of proposed scheme.

Based on the above research, to effectively enhance the efficiency and robustness of integration frequency band selection, this paper proposes the Adaptive Hoyer–L-moment Envelope Spectrum (AHLES) method. The technical workflow is illustrated in Figure 7, and the detailed procedure is as follows:

(1) Acquire modulated signals from ship-radiated noise.
(2) Using the fast SC algorithm [23], estimate the SCoh of the radiated noise by configuring an appropriate window length and a maximum cyclic frequency.
(3) Use the GRBD structure to divide the demodulation frequency bands along the spectral frequency axis f, obtaining a series of sub-band groups. Subsequently, a series of candidate EESs is constructed by integrating the SCoh magnitude over these spectrally partitioned sub-bands. Evaluate the richness of modulation information in each sub-band using the HL metric. Then, adaptively select the sub-bands using the golden section strategy.
(4) Calculate the envelope spectrum:
  (19) $$S_{x}^{AHLES}\left(\alpha \right)=\frac{1}{N}\sum_{j=1}^{N}\int_{f(j)}^{f(j)+\Delta f}{\left|\gamma_{x}\left(\alpha ,f\right)\right|}^{2}df$$
  where $S_{x}^{AHLES}\left(\alpha \right)$ denotes the adaptive Hoyer–L-moment envelope spectrum, *N* is the number of frequency bands in the sub-band $B_{j}(\sigma_{best})$, and f(j) represents the starting frequency of the sub-band $B_{j}(\sigma_{best})$.

## 4. Experimental Results and Performance

### 4.1. Simulation Analysis

**Figure 8:**
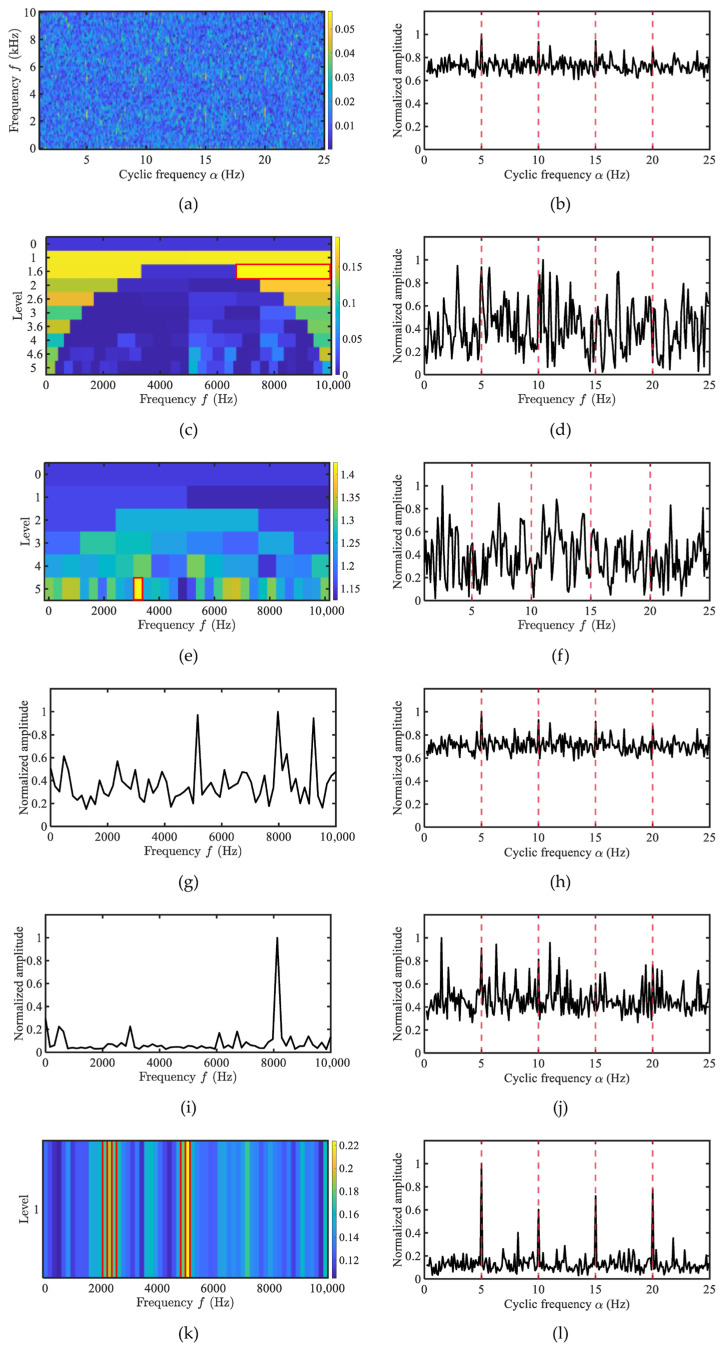
The comparison of demodulation methods for the simulated signal under Gaussian noise: (**a**) SCoh; (**b**) EES; (**c**) FK; (**d**) FK SES; (**e**) Autogram; (**f**) Autogram SES; (**g**) the weighting function of WEES; (**h**) WEES; (**i**) the weighting function of AWES; (**j**) AWES; (**k**) GRBD; (**l**) AHLES.

This section conducts a comparative analysis between the proposed AHLES method and conventional narrowband demodulation techniques (FK [8], Autogram [10]), as well as cyclostationarity-based approaches (EES, WEES [28], AWES [29]), using simulated signals. The simulated signal is consistent with that in Section 3.1, with the SNR set to −20 dB. The results are shown in Figure 8.

The red boxes in Figure 8 indicate the frequency bands selected using the AHLES method. As shown in Figure 8k, AHLES accurately selects both dual-carrier frequency bands, demonstrating its capability to capture modulation characteristics across multiple carrier bands. In contrast, both the FK and AWES methods select frequency bands above 7 kHz for demodulation, while the Autogram method only identifies the 2–3 kHz band. The weighted frequency bands in the WEES method are severely affected by noise, resulting in an inability to precisely locate the carrier bands. Under low-SNR conditions, AHLES correctly identifies the carrier frequency bands and exhibits stronger detection capability for modulated signals.

### 4.2. Calculation Cost

The computational efficiency of AHLES was evaluated against other methods using the signal described in Section 4.1. All tests were conducted in MATLAB R2022b on a platform with an AMD R7 processor and 32 GB of RAM. As shown in Table 1, the total execution time of AHLES is commensurate with that of established signal processing methods.

### 4.3. Performance Evaluation Using Monte Carlo Simulations

**Figure 9:**
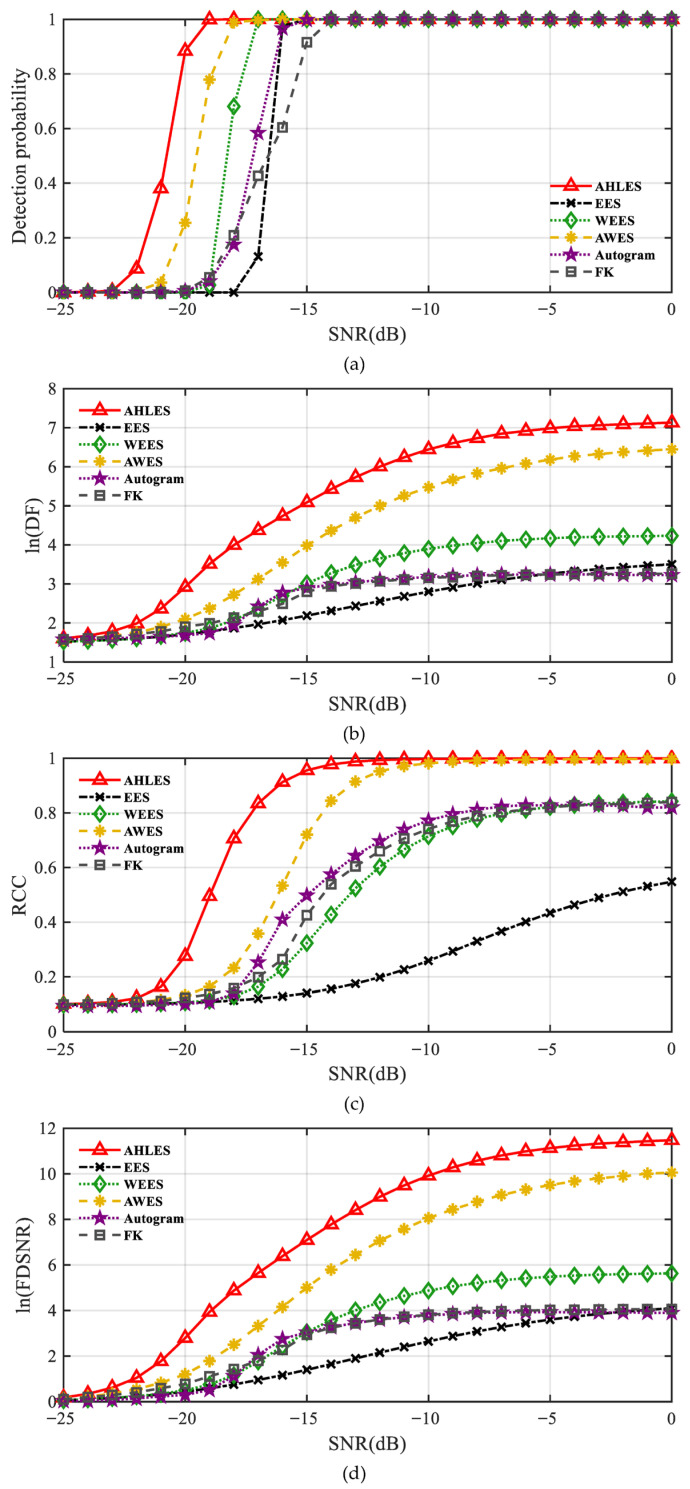
Monte Carlo simulations: (**a**) detection probability; (**b**) ln (DF); (**c**) RCC; (**d**) ln (FDSNR).

Monte Carlo experiments were conducted to validate the effectiveness and superiority of the proposed AHLES method under different SNR conditions, thereby compensating for the limitation of insufficient real data. The Average Peak-to-threshold Ratio (APR) detection method proposed by Weiqi Tong [29] was adopted to determine whether modulation features were successfully detected; modulation was considered to be identified when the metric $T_{APR}>1$. Three quantitative metrics, namely DF [27], RCC [32], and FDSNR [31], were selected to evaluate the SNR of the demodulated spectrum. The more significant the modulation-related features, the more prominent the amplitude of the modulation characteristic frequency and its harmonics in the spectrum, and thus the larger the ln(FDSNR), ln(DF), and RCC. The simulated signal is identical to that described in Section 3.1, with SNRs varying from −25 dB to 0 dB in 1 dB increments. The averages of the three quantitative metrics and the detection probability *P_d_* were computed via Monte Carlo simulation, where *P_d_* = *N_d_*/*N_total_*. Here, *N_d_* represents the number of trials when the modulation features are detected, and *N_total_* is the total number of trials, which is set to 1000. The experimental results are shown in Figure 9. Over the entire SNR range from −25 dB to 0 dB, AHLES consistently achieved the highest values across all three quantitative metrics and demonstrates superior detection probability compared to other methods. The Monte Carlo simulation results demonstrate that the proposed method outperforms other demodulation techniques under various SNR conditions.

### 4.4. Performance Evaluation Using Merchant Ship Data

For merchant ship data, the open-source hydroacoustic dataset named DeepShip [33] is selected. It contains 47 h and 4 min of real-world underwater recordings from 265 ships belonging to four categories, making it the largest openly available dataset of its kind. The signals in this dataset are sampled at 32 kHz.

Typical signals from four types of ships are chosen for demodulation comparison to verify the modulation feature extraction capability of the proposed method in complex underwater acoustic environments.

**Figure 10:**
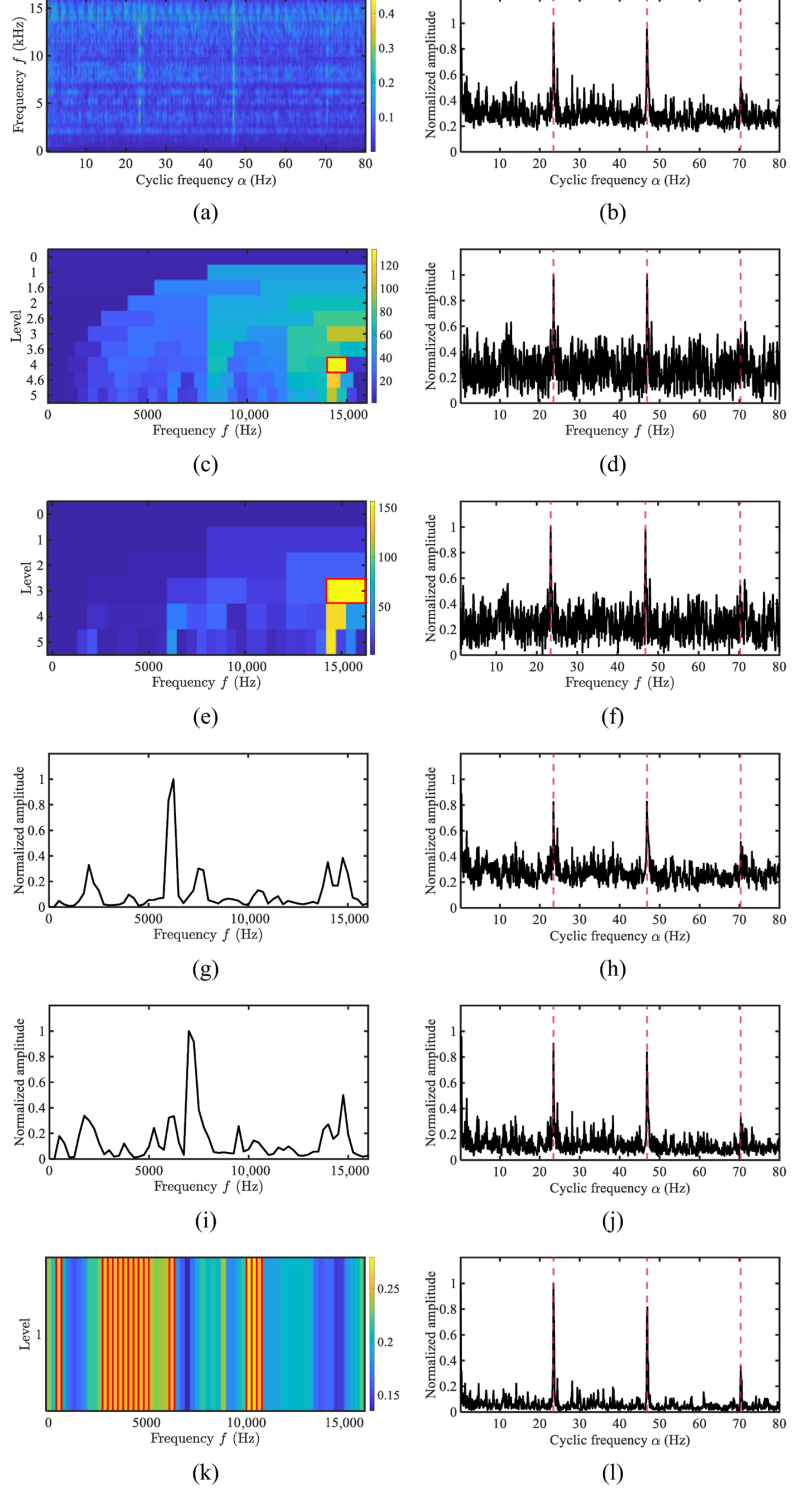
The comparison of demodulation methods for the three-blade propeller of cargo: (**a**) SCoh; (**b**) EES; (**c**) FK; (**d**) FK SES; (**e**) Autogram; (**f**) Autogram SES; (**g**) the weighting function of WEES; (**h**) WEES; (**i**) the weighting function of AWES; (**j**) AWES; (**k**) GRBD; (**l**) AHLES.

The demodulation spectra of a three-blade cargo ship are shown in Figure 10. The recorded rotational speed of the vessel was 1404 RPM, corresponding to a shaft rotation frequency of 23.4 Hz. The SCoh reveals significant interference in the signal. The AHLES method chooses frequency bands centered around 5 kHz for demodulation, while methods such as FK, Autogram, WEES, and AWES do not choose these bands. Consequently, the demodulated spectrum obtained by AHLES clearly reveals the fundamental frequency along with its second-order harmonics.

**Figure 11:**
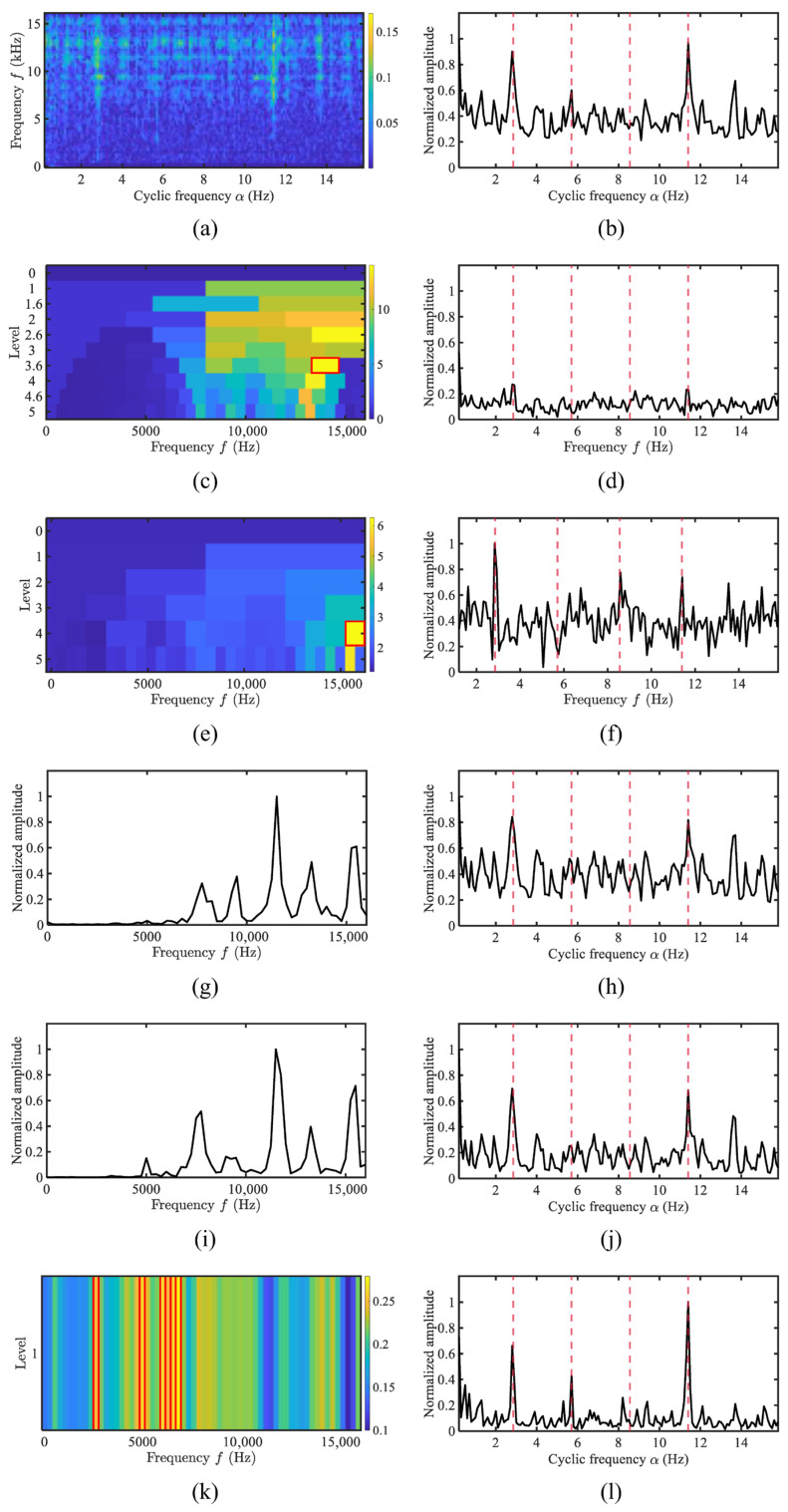
The comparison of demodulation methods for the four-blade propeller of passengership: (**a**) SCoh; (**b**) EES; (**c**) FK; (**d**) FK SES; (**e**) Autogram; (**f**) Autogram SES; (**g**) the weighting function of WEES; (**h**) WEES; (**i**) the weighting function of AWES; (**j**) AWES; (**k**) GRBD; (**l**) AHLES.

The demodulation spectra of a four-blade passenger ship are shown in Figure 11. The recorded rotational speed of the vessel was 168 RPM, corresponding to a shaft rotation frequency of 2.8 Hz. The fundamental frequency and third-order harmonics can be vaguely distinguished in the SCoh. Methods, including FK, Autogram, WEES, and AWES, select demodulation bands in higher frequency regions, failing to clearly reveal all harmonic curves. Only AHLES identifies the bands around 5 kHz, enabling the clear visualization of the fundamental frequency and multiple harmonics.

**Figure 12:**
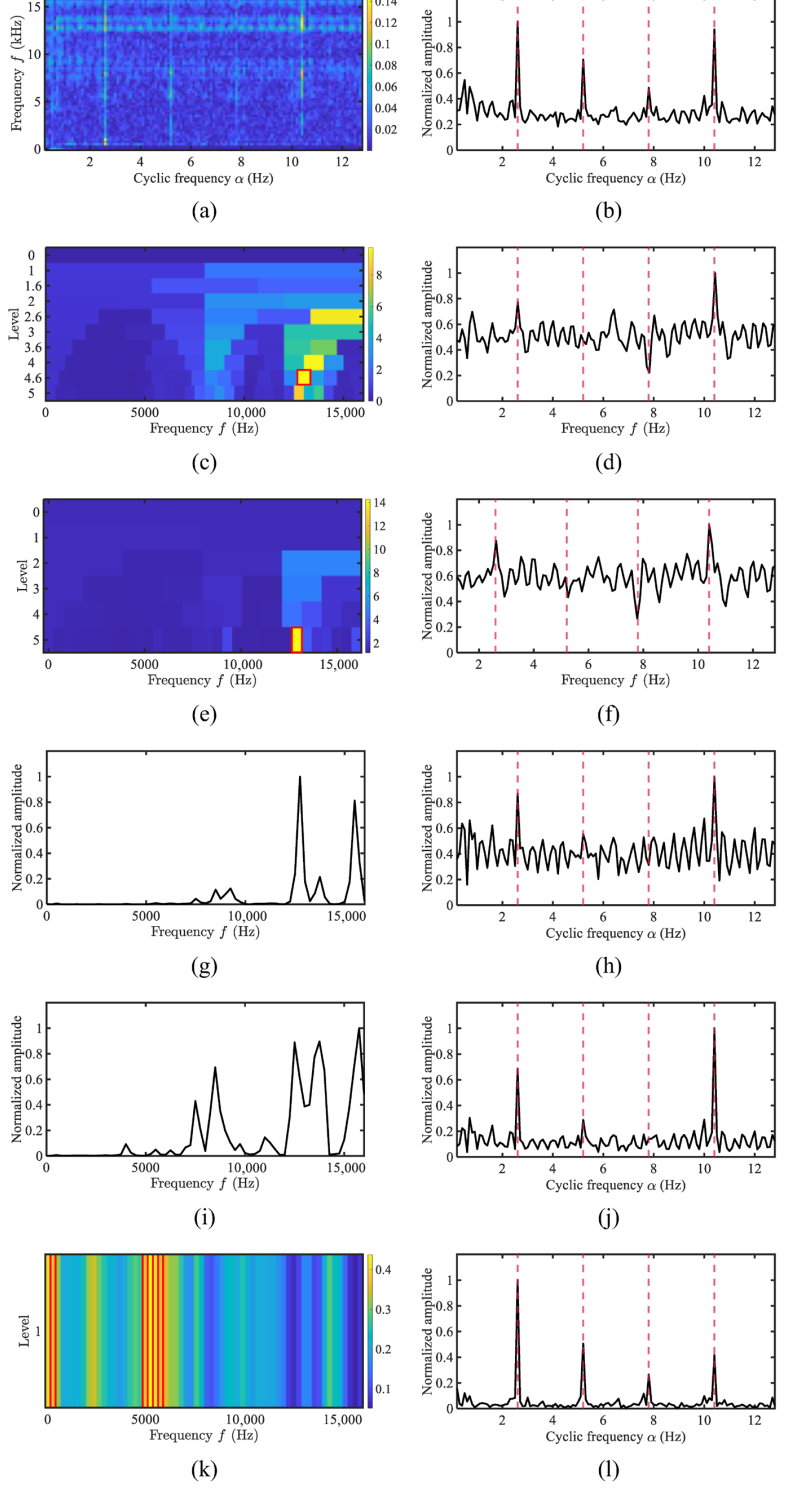
The comparison of demodulation methods for the four-blade propeller of tanker: (**a**) SCoh; (**b**) EES; (**c**) FK; (**d**) FK SES; (**e**) Autogram; (**f**) Autogram SES; (**g**) the weighting function of WEES; (**h**) WEES; (**i**) the weighting function of AWES; (**j**) AWES; (**k**) GRBD; (**l**) AHLES.

The demodulation spectra of a four-blade tanker are shown in Figure 12. The recorded rotational speed of the vessel was 156 RPM, corresponding to a shaft rotation frequency of 2.6 Hz. The fundamental frequency and third-order harmonics are partially discernible in the SCoh. Again, FK, Autogram, WEES, and AWES converge on mid-to-high frequency demodulation bands and cannot resolve the complete harmonic structure. In contrast, AHLES successfully localizes the frequency bands around 6 kHz, yielding a clear spectrum showing the fundamental frequency and fourth-order harmonics.

**Figure 13:**
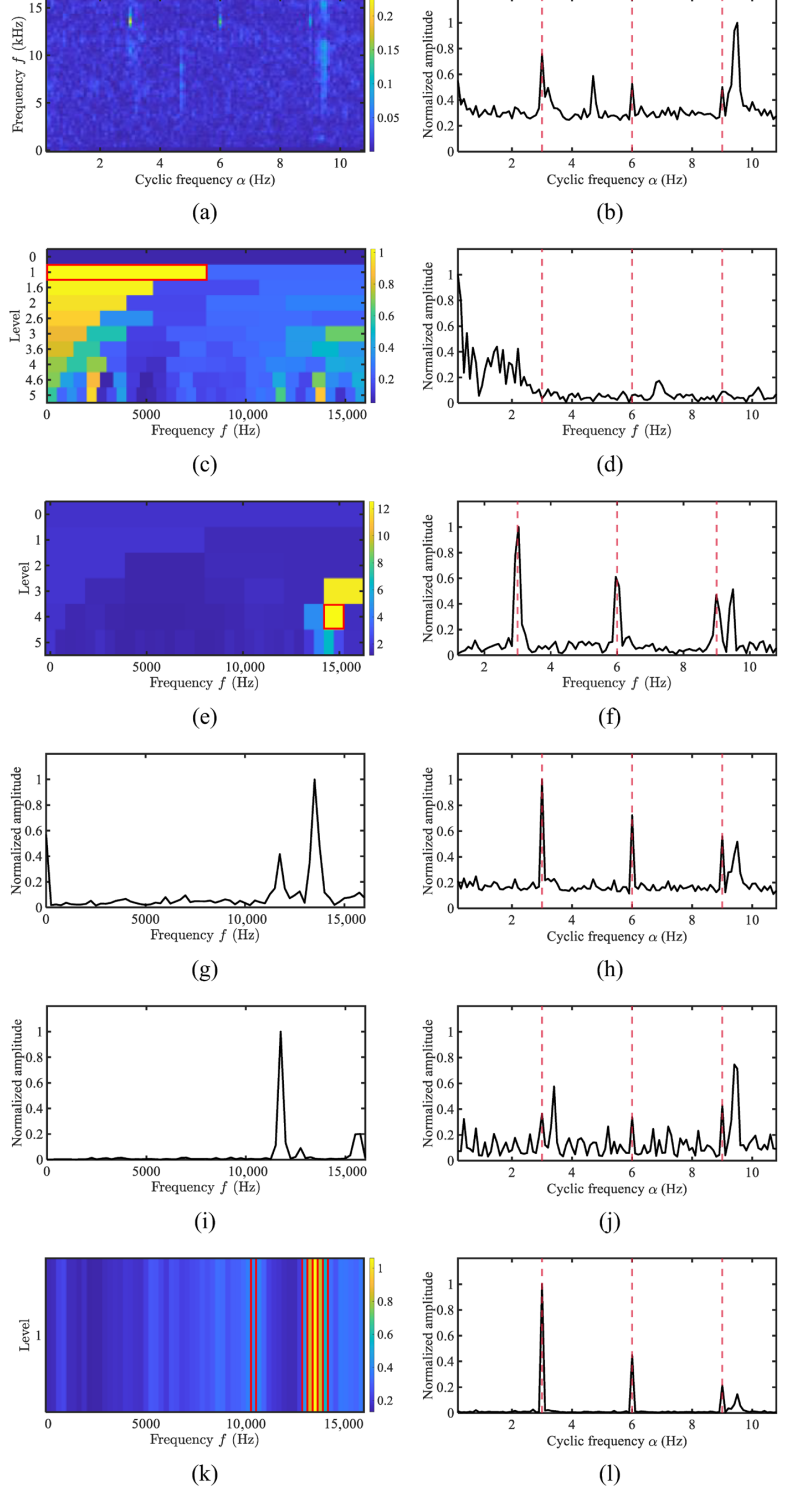
The comparison of demodulation methods for the three-blade propeller of Tug: (**a**) SCoh; (**b**) EES; (**c**) FK; (**d**) FK SES; (**e**) Autogram; (**f**) Autogram SES; (**g**) the weighting function of WEES; (**h**) WEES; (**i**) the weighting function of AWES; (**j**) AWES; (**k**) GRBD; (**l**) AHLES.

The demodulation spectra of a three-blade tug are shown in Figure 13. The recorded rotational speed of the vessel was 180 RPM, corresponding to a shaft rotation frequency of 3.0 Hz. The fundamental frequency and second-order harmonics are partially discernible in the SCoh. AHLES, Autogram, and WEES identify the carrier bands around 13 kHz, which clearly reveals the fundamental frequency and multiple harmonics. In contrast, methods including FK and AWES do not select those demodulation bands and thus fail to clearly reveal all harmonic curves. Since AHLES localizes the narrowest frequency band, it achieves optimal demodulation performance.

Both simulation analysis and real-data experiments demonstrate that the AHLES method achieves superior demodulation performance compared to conventional techniques. The method remains effective even in low-SNR scenarios and provides a robust, prior-knowledge-free demodulation tool for ship target recognition with strong anti-interference capability.

### 4.5. Quantitative Comparison of the Proposed Method and Typical Methods

**Figure 14:**
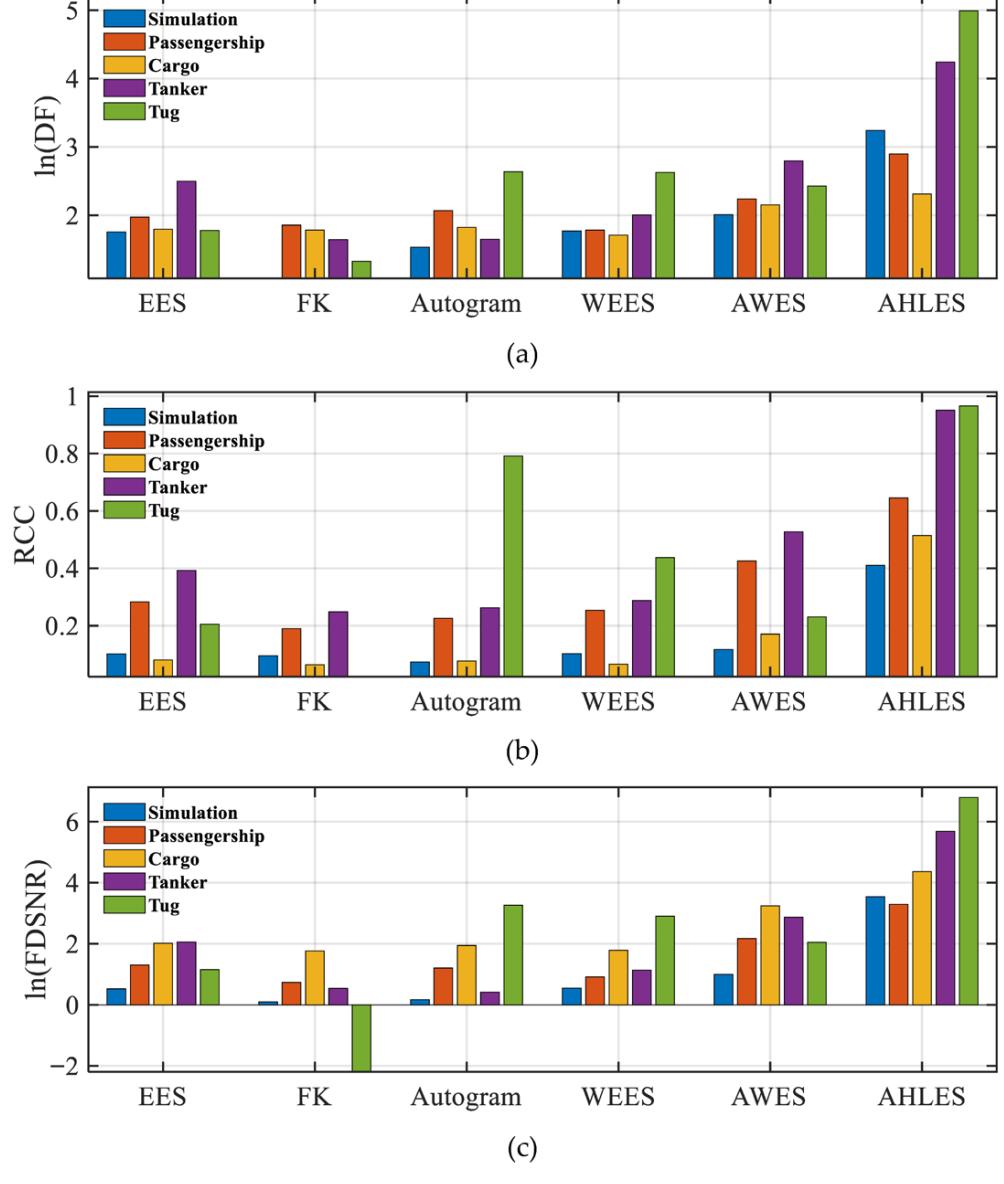
Quantitative indicators achieved by the proposed method and typical methods on simulated and merchant ship signals: (**a**) ln(DF); (**b**) RCC; (**c**) ln(FDSNR).

A quantitative analysis was conducted to further evaluate and compare the demodulation performance of the proposed method against other methods. Figure 14 depicts the three quantitative indicators obtained by the proposed method and the selected competing methods on both simulated and merchant ship signals. The AHLES method achieves significantly higher values across all three metrics on the simulation and four merchant ship signals compared to the five other methods, further verifying its effectiveness and superiority in demodulation performance.

## 5. Conclusions

To address the challenge of extracting weak modulation features from ship targets in low signal-to-noise ratio (SNR) environments, this paper proposes an Adaptive Hoyer–L-moment Envelope Spectrum (AHLES) method. The approach constructs an HL indicator to quantify the richness of modulation information and employs a golden section strategy to adaptively select the optimal integration frequency band. The effectiveness of AHLES is evaluated through comprehensive comparisons with five state-of-the-art demodulation techniques—EES, FK SES, WEES, AWES, and Autogram—using both simulated signals and merchant ship data. Experiment results based on the DeepShip dataset demonstrate that AHLES consistently identifies blade-related modulation characteristics with multi-carrier frequency band distributions across diverse vessel types. Furthermore, it achieves superior performance across multiple quantitative evaluation metrics. These results validate that AHLES is a robust and versatile demodulation technique capable of operating effectively across various SNR scenarios. Notably, it provides a prior-knowledge-free and highly interference-resistant solution for ship target recognition in practical underwater acoustic applications. Future work will include collecting vibrations and communication datasets to systematically validate the method’s effectiveness and superiority in these fields, thereby expanding its application boundaries.

## Figures and Tables

**Table 1:** Comparison of calculation time.

Method	EES	FK	Autogram	WEES	AWES	AHLES
Calculation time (s)	0.090924	0.071979	2.063884	0.100143	0.091279	0.125408

## Data Availability

The DeepShip dataset is publicly available at https://github.com/irfankamboh/DeepShip (accessed on 10 September 2024).
